# Nasolacrimal intubation in transcanalicular endoscopic dacryoplasty: a long-term follow-up study

**DOI:** 10.1038/s41598-023-34351-0

**Published:** 2023-05-09

**Authors:** Julian Alexander Zimmermann, Eliane Luisa Esser, Ralph-Laurent Merté, Moritz Fabian Danzer, Friederike Rosenberger, Viktoria C. Brücher, Nicole Eter, Maged Alnawaiseh, Alina Friederike Blumberg, Larissa Lahme, Natasa Mihailovic

**Affiliations:** 1 Department of Ophthalmology, University of Muenster Medical Center, Domagkstraße 15, 48149 Muenster, Germany; 2 Institute of Biostatistics and Clinical Research, University of Muenster, Muenster, Germany; 3 Department of Ophthalmology, Klinikum Bielefeld Gem. GmbH, Bielefeld, Germany; 4 Department of Ophthalmology, Klinikum Fulda gAG, University of Marburg, Campus Fulda, Fulda, Germany

**Keywords:** Lacrimal apparatus diseases, Eye diseases

## Abstract

Nowadays, transcanalicular endoscopic dacryoplasty represents the majority of lacrimal duct surgery procedures performed in adults in specialised centers. However, there are still hardly any data available regarding the intra- and postoperative care, particularly regarding the duration of silicone tube intubation (STI). Our aim was to evaluate the relation between tube duration and recurrence of symptoms in patients who underwent transcanalicular microdrill dacryoplasty (MDP) in a long-term setting. Medical records of 576 adult patients after MDP were retrospectively reviewed. A total of 256 eyes of 191 patients could be included. The median follow-up time was 7.83 [7.08; 9.25] years. In 57.0% of the cases there was still full resolution of symptoms at the time of the survey. The median duration of the STI was 6 [3.00; 6:00] months. When distinguishing between a tube duration < 3 months and ≥ 3 months there was a significant difference in the long-term success rate (< 3 months: 38%; ≥ 3 months: 61%; p = 0.011). In conclusion, an early removal of the STI (< 3 months) after transcanalicular MDP seems to be associated with a higher incidence of recurrence of symptoms. This should be considered in the intra- and postoperative care of patients following this minimally invasive first-step procedure.

## Introduction

Besides tear film disorders and eyelid abnormalities nasolacrimal duct obstruction (NLDO) is the most common reason for epiphora in adults in ophthalmologic practice^1^. NLDO can be primary or secondary acquired. The etiopathogenesis of primary acquired nasolacrimal duct obstruction (PANDO) seems to be multifactorial and is attributed to unspecific chronic fibrosing inflammation, vascular congestion and mucosal edema, whereas secondary acquired nasolacrimal duct obstruction (SANDO) occurs after infections, trauma, irradiation or neoplasia^2–4^. While in some cases a lacrimal drainage system (LDS) irrigation or anti-inflammatory treatment for underlying inflammation of the LDS is sufficient to resolve the obstruction, in a large proportion of patients nasolacrimal duct (NLD) surgery is required^5^.

Dacryocystorhinostomy (DCR) is currently still defined as the gold standard for the treatment of NLDO in international literature^6^. Both, endonasal DCR (EN-DCR) and external DCR (EXT-DCR), show very good long-term success rates of approximately 90% and current data do not allow any clear conclusions to be drawn regarding the superiority of one approach to the other^6^. Nevertheless, both EN-DCR and EXT-DCR, represent invasive bypass surgery that do not preserve the anatomy of the LDS. However, technological progress in endoscopic medical technology have enabled the establishment of minimally invasive, anatomy-preserving procedures in nasolacrimal duct surgery.

Thus, in recent years the treatment options for NLDO have improved significantly due to transcanalicular microendoscopic techniques which are already used as first-step procedures in specialized centers in Germany^7,8^. Further advantages of these procedures compared to DCR are the short operative time and the shorter postoperative recovery.

In particular, transcanalicular microdrill dacryoplasty (MDP) or transcanalicular laser dacryoplasty (LDP) have significantly decreased the number of EXT-DCRs performed in tertiary eyecare centers specialised in nasolacrimal duct surgery^8^. In contrast to diode laser assisted LDP, MDP is performed using a microdrill with a diameter of 0.38 mm and 6000 rpm for recanalisation. It is suitable for opening total and subtotal intra- and postsaccal obstructions as well as for removing membranes, folds and polyps as well as for crushing dacryoliths. However, the contraindications for these minimally invasive techniques must always be kept in mind: in the case of long-segmented (particularly canalicular) obstructions, mucoceles, acute dacryocystitis, and complex midface fractures, microendoscopic recanalization should be avoided^7,8^.

The postoperative success rate after three months ranges between 75 and 84%^9,10^. Long-term data of a large cohort after transcanalicular MDP have been published recently for the first time and show a success rate of about 60% after a follow-up period of 8.7 ± 0.9 years^11^.

While these minimally invasive techniques represent a first-step procedure for patients with PANDO in Germany, there are still hardly any long-term or prospective data on these techniques in the literature. Particularly, there is also a lack of data regarding the duration of postoperative silicone tube intubation following these procedures. Nasolacrimal duct surgery experts usually recommend a tube duration of approximately 3 to 6 months after recanalization by transcanalicular MDP in order to avoid postoperative adhesions^7,8^. Currently, there is no clinical evidence from a study to support this recommendation for endoscopic dacryoplasties like transcanalicular MDP. As the number of minimally invasive nasolacrimal duct surgery procedures will certainly further increase in the future, more clinical data is required for optimising patient care regarding these modern procedures.

Therefore, the aim of this retrospective long-term study was to compare the success rates of transcanalicular MDP in relation to the silicone tube intubation duration in a large cohort in order to be able to give a reliable recommendation regarding the intubation duration and thereby improve postoperative patient care and success rates after transcanalicular endoscopic dacryoplasty.

## Material and methods

A retrospective analysis of 576 medical records of adult patients who underwent transcanalicular MDP due to PANDO as a first procedure in at least one eye at the Department of Ophthalmology of the University of Muenster Medical Center in the period from 01/2009 to 12/2011 was performed and all patients with available data on tube intubation duration and recurrence were included. Patients who intraoperatively were found to have other causative pathology like intrasaccal granulomas and synechiae were excluded from the analysis. The study was approved by the Ethics Committee and complied with the principles of the Declaration of Helsinki.

The data on silicone tube intubation duration was obtained either from the medical record or by a telephone interview, in case the tube removal had not been performed at our department. Data on the recurrence of symptoms in these patients were also assessed by a telephone survey in the period from 2017 to 2019. In this study, only the complete resolution of symptoms ("no epiphora") without subsequent dacryocystitis, inflammation of the lacrimal apparatus or any additional surgical intervention during the follow-up period was defined as success, corresponding to a Munk score of grade 0^11,12^.

Data were collected in Microsoft Office Excel spreadsheet software version 2206 (Microsoft, Redmond, WA, USA). Since the data did not fit a normal distribution descriptive data are presented as median [25th-; 75th-percentile]. To determine statistical significance between different groups of intubation duration, the groups were compared with Generalized Estimating Equations (GEEs) to account for the within-subject correlation of measurements using R version 4.1.2. An exchangeable correlation structure was chosen, and p-values were computed by Wald-Tests using the robust variance estimators for GEEs. The chosen level of statistical significance was p < 0.05.

### Surgical procedure of transcanalicular MDP

**Figure 1:**
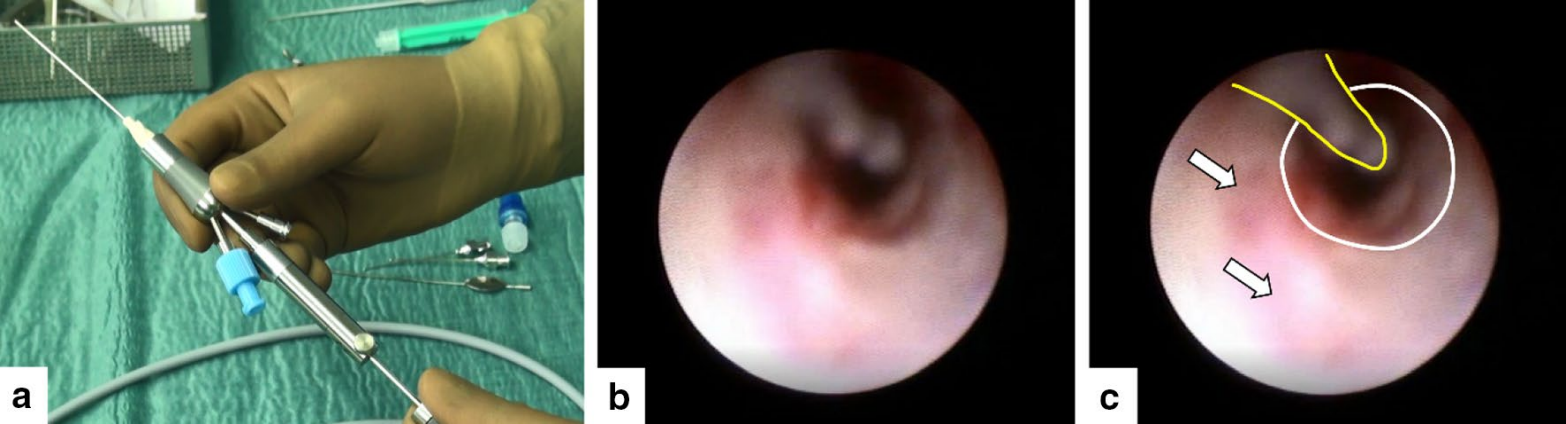
Endoscopic transcanalicular microdrill dacryoplasty (MDP). (**a**) Design of the modular microendoscope (outside diameter 1.1 mm, Polydiagnost GmbH, Hallbergmoos). (**b,c**) Momentary snapshot during a transcanalicular MDP showing the microdrill at the 11 o’clock position (yellow marking), the distal nasolacrimal duct (white circle) as well as erosions of the ductonasolacrimal mucosa with partly bloody, partly whitish coatings in the foreground (white arrows).

**Figure 2:**
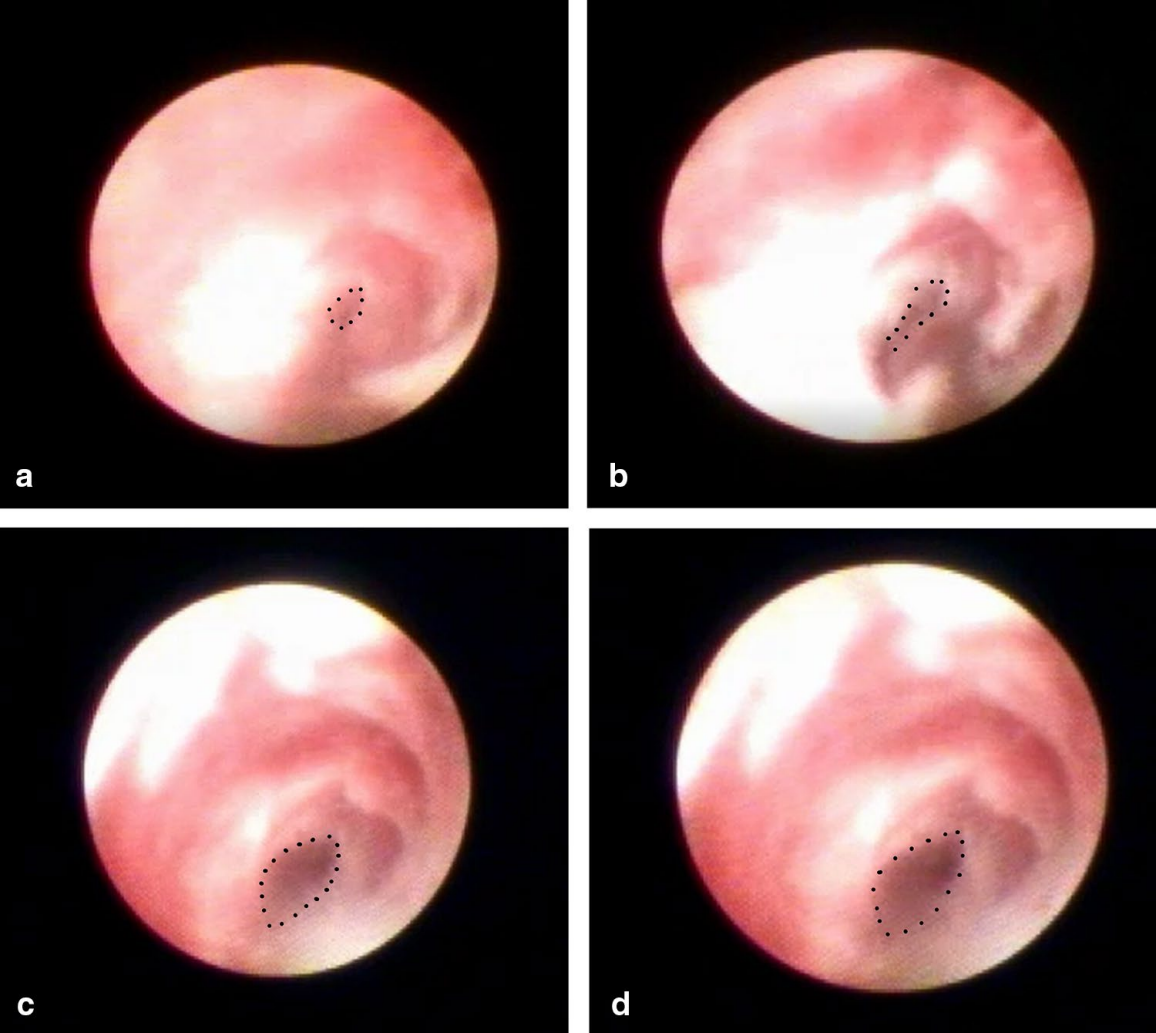
Intraluminal view of the site of nasolacrimal duct obstruction during treatment with transcanalicular microdrill dacryoplasty. (**a–d**) represent the chronological image sequence with enlargement of the opening after application of the microdrill (dotted black line).

**Figure 3:**
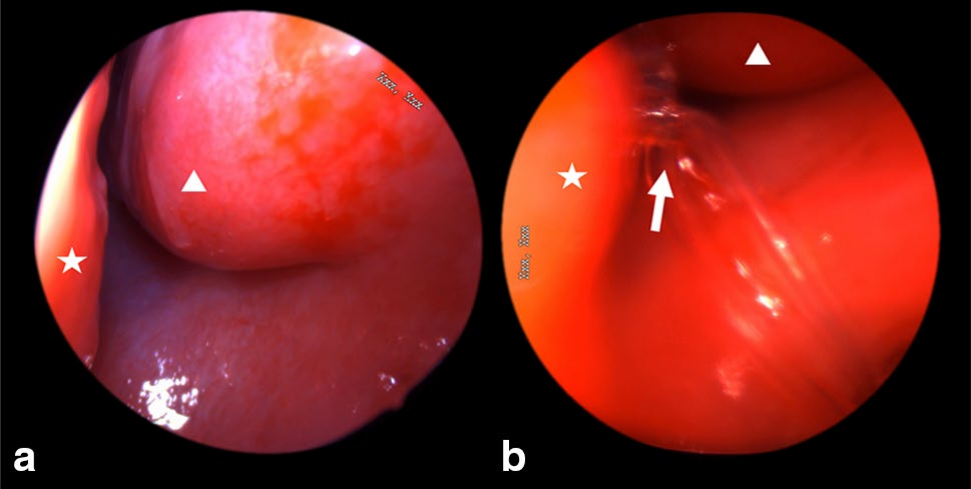
Endonasal endoscopic view of the left inferior turbinate (arrowhead) and septum (star) before (**a**) and after (**b**) silicone tube intubation showing the exit point of the tube just below the inferior turbinate (white arrow).

The procedure is usually performed under general anesthesia. After dilation of the lacrimal puncta and canaliculi by an experienced nasolacrimal duct surgeon using a conical probe and/or a Jünnemann probe, the modular three-port, single-channelled endoscope (outer diameter of the PolyShaft® 1.1 mm, PolyDiagnost GmbH, Hallbergmoos, Germany) including a microdrill (diameter: 0.38 mm, PolyDiagnost GmbH, Hallbergmoos, Germany) and a semi-rigid fiber optic (diameter: 0.55 mm, resolution: 6000 pixels, field of view: 70°, Polydiagnost GmbH, Hallbergmoos, Germany) is then inserted over the superior or inferior lacrimal punctum to the medial wall of the lacrimal sac until contact with the underlying lacrimal bone. The endoscope is rotated vertically and advanced into the nasolacrimal duct or to the site of obstruction, respectively. By using the three-port-endoscope and the PolyShaft® of 1.1 mm precise drilling is ensured due to simultaneous visualisation of the site of obstruction. Irrigation should be continuous to obtain a clear endoscopic image. By retracting the endoscope, the nasolacrimal duct, the lacrimal sac and the canaliculus can be assessed (Fig. 1). After locating the obstruction in the nasolacrimal duct (e. g. due to membranes, mucus, dacryoliths), recanalization is performed by pushing the microdrill out of the channel and activating it with the foot pedal (Fig. 2). After completion of endoscopy bicanalicular intubation with a silicone tube according to the atraumatic Münster intubation technique is performed (Fig. 3)^13^. At the end of the procedure as well as for two weeks postoperatively, antibiotic (Floxal^®^ EDO, Ofloxacin, 3 mg/ml, q.i.d.) and astringent (Berberil^®^, Hypromellose 3.2, mg/ml, t.i.d) eye and nasal drops (Nasivin^®^, Oxymetazoline Hydrochloride, 0.1 mg/ml, t.i.d.) are applied.

### Research ethics approval

All procedures performed in studies involving human participants were in accordance with the ethical standards of the institutional and/or national research committee and with the 1964 Helsinki declaration and its later amendments or comparable ethical standards. The study was approved by the Ethics Committee of the University of Muenster, North Rhine Westphalia, Germany (2015-532-f-S). Informed consent was waived by the Ethics Committee of the University of Muenster, North Rhine Westphalia, Germany due to the retrospective nature of the study.

## Results

A total of 256 eyes of 191 patients who had undergone transcanalicular MDP and in whom the duration of silicone tube intubation and recurrence data were known could be included. The median follow-up time was 7.83 [7.08; 9.25] years. 126 (66.3%) patients were female, 64 (33.7%) male. The median age was 63.00 [51.00; 71.00] years. The majority of the study population was Caucasian (252 eyes, 98.4%), one patient was Asian (2 eyes, 0.8%) and one patient was Hispanic (2 eyes, 0.8%). Sixty-five (34.2%) patients received bilateral transcanalicular MDP and 126 (66.3%) patients received unilateral transcanalicular MDP. Overall, 146 of the 256 cases (57.0%) were still symptom-free at the time of the follow-up. The preoperative and/or intraoperative irrigation findings showed a partial obstruction in 129 cases (50.4%) and a complete obstruction in 90 cases (35.2%). In 37 cases (14.4%) the grade of obstruction could retrospectively not be determined from the medical records. Seventy-six of the 129 cases with partial obstruction (58.9%) and 49 of the 90 cases with complete obstruction (54.4%) were still symptom-free at the time of the follow-up. The demographic data are summarised in Table 1.

**Table 1:** Demographics and characteristics of the study population.

Patients/eyes (n)	190/256
Age in years	63.00 [51.00; 71.00]
Gender (f/m)	126 (66%)/64 (34%)
Unilateral/bilateral symptoms	65 (34%)/126 (66%)
Follow-up time in years	7.83 [7.08; 9.25]
Symptom-free at follow-up	146 (57.0%)
Duration of tube intubation in months	6 [3.00; 6:00]

Data are presented as median [25th-; 75th-percentile].

*f* female; *m* male.

In 45 cases (18%) the duration of tube intubation was less than 3 months, in 66 cases (26%) between 3 and < 6 months, in 140 cases (55%) between 6 and < 12 months, and in five cases (1%) more than 12 months. Spontaneous tube dislocation leading to tube removal occurred in 9 of the 256 cases (3.5%), of which six occurred within the first 3 months and led to an early tube removal. Other reasons for tube removal before 3 months were the patients' scheduling preferences and tube removal by an ophthalmologist outside our clinic.

Inflammatory complications occurred in a total of eight cases (3.1%) and included a subsequently experienced dacryocystitis in three cases (1.2%) as well as the occurrence of canaliculitis (n = 1, 0.4%) and pyogenic granuloma (n = 1, 0.4%) in one case each. Non-specified inflammation of the lacrimal apparatus was described in three cases (1.2%). In another case, remaining tube remnants led to a consecutive inflammation of the LDS requiring a second surgical procedure (n = 1, 0.4%). Overall, additional surgical procedures became necessary in 48 cases (18.8%). Details of these second procedures are shown in Table 2.

**Table 2:** Detailed presentation of the cases requiring additional surgical intervention.

	n (%)
Rate of recurrence	110 of 256 (43.0%)
Second surgical procedure	48 of 110 (43.6%)
Transcanalicular MDP	26 of 48 (54.2%)
External dacryocystorhinostomy	17 of 48 (35.4%)
Canaliculotomy	1 of 48 (2.1%)
Unknown intervention (carried out elsewhere)	4 of 48 (8.3%)

*MDP* microdrill dacryoplasty*.*

**Figure 4:**
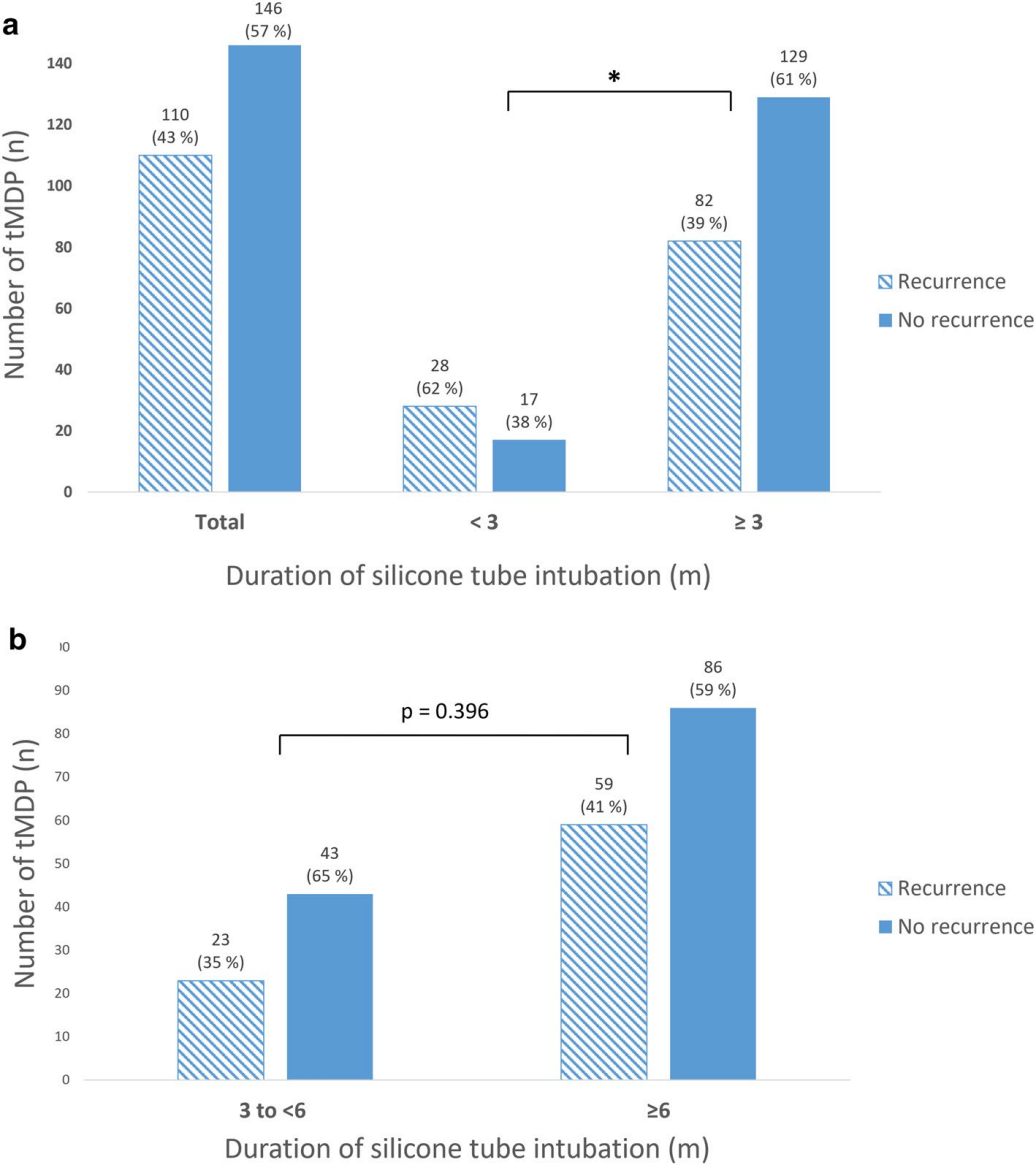
Recurrence rates after transcanalicular microdrill dacryoplasty (tMDP) of our study group by duration of silicone tube intubation in months (m). (**a**) Distribution of recurrence in patients with an intubation duration of < 3 and ≥ 3 months. (**b**) Distribution of recurrence in patients with an intubation duration from 3 to < 6 and ≥ 6 months. *p < 0.05.

In 38% of cases with a tube duration of < 3 months and in 61% of cases with a tube duration of ≥ 3 months, there was still no recurrence of symptoms at the time of follow-up (Fig. 4a). The difference in the success rate between these two groups was statistically significant (p = 0.011). A subgroup analysis of the cases with a duration of tube intubation from 3 to < 6 months and ≥ 6 months showed no significant difference between these two groups (p > 0.05, Fig. 4b).

## Discussion

In the present long-term follow-up study, we demonstrated that silicone tube intubation after transcanalicular MDP of less than 3 months was significantly more frequently associated with recurrence. To our knowledge, the present study is the first to evaluate the relationship between silicone tube intubation duration and recurrence in patients undergoing transcanalicular dacryoplasty over such a long follow-up period.

Our results confirm the widespread expert opinion that a tube intubation of at least 3 months is recommended for transcanalicular recanalizing microendoscopic procedures, particularly microdrill dacryoplasty. Intubating over a longer period of time, however, does not seem to show any further benefit and might also lead to biofilm formation. Pathophysiologically, endoscopic recanalization is thought to cause perioperative micro-injuries to the nasolacrimal mucosa itself, which would otherwise result in scarring and adhesions during the healing process in the absence of intubation, leading to re-obstruction.

The major advantages of minimally invasive methods represent endoductolacrimal visualization, the possibility of classifying the type of obstruction as well as sight-controlled recanalization and preservation of all involved structures of the lacrimal apparatus. The risk of complications and bleeding is significantly reduced compared to DCR^7^. However, a disadvantage compared to DCR is the reduced long-term success rate. While long-term data on DCR show a success rate of around 90%, the only publication with a similar follow-up period after transcanalicular dacryoendoscopy showed a success rate of just under 60%, although the initial success rate was 84%^11^. When comparing success rates of different studies on nasolacrimal duct surgery, the definition of success and the careful and correct indication for the performance of microendoscopic procedures need to be reviewed critically^11^. In our study only a complete resolution of epiphora (corresponding to a Munk score of grade 0) was defined as success.

Moreover, the pathogenesis of PANDO, the main indication for endoscopic procedures, is still not yet fully understood. It may be assumed that due to chronic inflammation and vascular congestion resulting in fibrous obstruction a minimally invasive procedure might not prevent a re-obstruction over the years. The pathophysiology of so-called functional obstruction, i.e. epiphora despite anatomically free irrigation, has also not been clarified with certainty and could have contributed to the increased incidence of the reported recurrence^14–16^.

Lee et al. also showed that the type of obstruction has significant influence on the success rate^17^. They included 156 patients undergoing lacrimal duct endoscopy and distinguished between PANDO with secretory (mucus, stones, granulation) and structural (membrane) obstruction. The success rate after 6 months was significantly higher for the structural (95.3%) than for the secretory type of obstruction (79.9%, p = 0.001). Success was defined as a Munk score of 0 to 1 with concomitant tear film meniscus height of < 300 µm and a passed irrigation test after tube extubation. No microdrill or laser was used in this study, so it can be speculated to what extent recanalization by microdrill or laser has an advantage over "blunt" recanalization with the endoscopic probe. For this purpose further studies are desirable in order to aim for a uniform approach of treating NLDO.

There are several limitations of the present study worth noting. First, our study has a retrospective design, therefore we cannot subsequently distinguish between PANDO with either secretory or structural obstructions and comment on the possibly different outcomes. Nevertheless, our patient population is quite homogeneous since we only included patients with PANDO and without previous lacrimal duct surgery. Second, differences in the occurrence of recurrence may also be due to demographic, behavioral, and clinical differences between patients because of the absence of a standardized protocol during the follow-up period. A prospective, randomized, study design could provide clarity. Further studies with a prospective design are needed for this purpose. Furthermore, no conclusions can be drawn regarding the anatomical patency of the LDS, since we did not perform irrigation of the LDS at the time of the follow-up. However, measuring success by patient satisfaction and resolution of symptoms is a widely used tool in the evaluation of nasolacrimal duct surgery and the most important factor for patient satisfaction^18,19^.

In conclusion, our results suggest that an intubation duration with a silicone tube of less than 3 months might lead to a higher incidence of recurrence in patients after transcanalicular MDP. Taken these findings into account and carefully considering the indications including the type of obstruction, endoscopic dacryoplasty is a minimally invasive procedure which is well suited as a first-step procedure.

## Data Availability

The datasets used and/or analysed during the current study available from the corresponding author on reasonable request.
